# Predicting Influenza Antigenicity by Matrix Completion With Antigen and Antiserum Similarity

**DOI:** 10.3389/fmicb.2018.02500

**Published:** 2018-10-23

**Authors:** Peng Wang, Wen Zhu, Bo Liao, Lijun Cai, Lihong Peng, Jialiang Yang

**Affiliations:** ^1^College of Information Science and Engineering, Hunan University, Changsha, Changsha, China; ^2^School of Mathematics and Statistics, Hainan Normal University, Haikou, China; ^3^School of Computer Science, Hunan University of Technology, Zhuzhou, China; ^4^Icahn Institute for Genomics and Multiscale Biology, Icahn School of Medicine At Mount Sinai, New York, NY, United States

**Keywords:** hemagglutination inhibition assay, low-rank matrix completion, influenza antigenicity, antigenic map, HA protein sequence information

## Abstract

The rapid mutation of influenza viruses especially on the two surface proteins hemagglutinin (HA) and neuraminidase (NA) has made them capable to escape from population immunity, which has become a key challenge for influenza vaccine design. Thus, it is crucial to predict influenza antigenic evolution and identify new antigenic variants in a timely manner. However, traditional experimental methods like hemagglutination inhibition (HI) assay to select vaccine strains are time and labor-intensive, while popular computational methods are less sensitive, which presents the need for more accurate algorithms. In this study, we have proposed a novel low-rank matrix completion model MCAAS to infer antigenic distances between antigens and antisera based on partially revealed antigenic distances, virus similarity based on HA protein sequences, and vaccine similarity based on vaccine strains. The model exploits the correlations of viruses and vaccines in serological tests as well as the ability of HAs from viruses and vaccine strains in inferring influenza antigenicity. We also compared the effects of comprehensive 65 amino acids substitution matrices in predicting influenza antigenicity. As a result, we applied MCAAS into H3N2 seasonal influenza virus data. Our model achieved a 10-fold cross validation root-mean-squared error (RMSE) of 0.5982, significantly outperformed existing computational methods like antigenic cartography, AntigenMap and BMCSI. We also constructed the antigenic map and studied the association between genetic and antigenic evolution of H3N2 influenza viruses. Finally, our analyses showed that homologous structure derived amino acid substitution matrix (HSDM) is most powerful in predicting influenza antigenicity, which is consistent with previous studies.

## Introduction

According to the United States Centers for Disease Control and Prevention (CDC), seasonal influenza and its linked respiratory diseases cause approximately 650,000 deaths annually worldwide, posing a serious threat to human health and socio-economic environment (WHO, 2017). This is mainly attributed to seasonal influenza viruses that frequently evade immunity in the human population through mutations in their hemagglutinin (HA) and neuraminidase (NA) surface glycoproteins (Hay et al., 2001; Neher et al., 2016). The most effective way to prevent influenza virus infection is to inoculate vaccines with similar antigenicity to the influenza virus (Sun et al., 2013). Therefore, timely and accurate identification of the effectiveness of existing vaccines on circulating virus strains is critical for vaccine design and influenza surveillance (Smith et al., 2004; Huang et al., 2017). However, the task is challenging (Blackburne et al., 2008; Yao et al., 2017). To facilitate the selection and design of vaccine strains, the World Health Organization's (WHO) Global Influenza Surveillance and Response System (GISRS) continuously monitors genotypic and antigenic characteristics of circulating viruses (Barr Ig, 2014).

The hemagglutination inhibition (HI) is one of the most popular experimental methods for measuring the effectiveness of a vaccine against an influenza virus (Hirst, 1943). It is a binding assay used to characterize the ability of antisera (vaccines) to block HA of antigens (viruses) from agglutinating red blood cells (RBC). Based on the HI assay, the concept of antigenic distance can be used to quantitatively describe the closeness among antigens. The antigenic distance is often defined to be the Euclidean distance between their representing vectors in a normalized HI table according to multiple reference antisera (Cai et al., 2010). HI assay and its derived antigenic distance provide great convenience for comparing antigenic similarity among influenza viruses (Fouchier et al., 2010; Neher et al., 2016). However, HI assays are expensive and time-consuming, so it is impractical to use it to measure the antigenic similarity among all antigens and antisera (Sun et al., 2013). This urges the need to explore effective computational methods (Liao et al., 2012a,b; Chen et al., 2018) to estimate the antigenic distance between an antigen and an antiserum (Liao et al., 2010, 2015a,b; Li et al., 2013; Liang et al., 2016; Peng et al., 2017).

A popular category of methods for predicting the antigenicity of influenza virus is the sequence-based method. Unlike imputation-based methods, sequence-based methods often explore the association between mutations in the HA protein and antigenic distances obtained from serological tests (Lee and Chen, 2004; Barnett et al., 2012; Li et al., 2016). The antigenic difference between two influenza viruses indicates whether they antigenic variant, which is measured by either an antigenic distance or simply a binary value (Lee and Chen, 2004; Smith et al., 2004; Liao et al., 2008). For example, a model based on multiple logistic regression was proposed by Liao et al. to predict antigen variants. For further exploration, 65 amino acid substitution models based on 20 amino acid physicochemical groups were also studied. The experimental results showed that high agreement was achieved in the H3N2 influenza data from 1999 to 2003 (Liao et al., 2008). Huang et al. introduced a decision tree algorithm to predict antigenic variants (Huang et al., 2009). Sun et al. proposed a bootstrapped ridge regression model consisting of antigenic related sites, which uses the quantitative amino acid substitutions in the HA1 [a sub-unit of HA forming globular domain (Wang et al., 2015)] protein sequence to predict antigenic distances (Sun et al., 2013). Inspired by the co-evolution of HA1 that may have contributed to antigen evolution, Yang et al. integrated the single mutation and co-mutation characteristics of the HA1 sequence and proposed a Lasso model (Yang et al., 2014). Neher et al. proposed an optimization model for interpreting known antigen data and studied its ability to predict future influenza virus population composition (Neher et al., 2016). However, these methods rely on the reliability of rapidly changing antigen-associate sites (Sun et al., 2013).

Imputation-based methods are widely used for predicting and visualizing the antigenicity of influenza viruses (Smith et al., 2004; Cai et al., 2010; Barnett et al., 2012). They are based on the assumption that the antigens and antisera are located in a low dimensional space (i.e., the normalized HI table is of low rank), so the HI table can be fully recovered from partially revealed HI titers (Lapedes and Farber, 2001). For example, Smith et al. proposed antigenic cartography for visualizing and predicting antigenic evolution of influenza viruses (Smith et al., 2004). They first transformed the known values in the HI table to Euclidean distances and then embedded them into a 2D map using the modified multidimensional scaling (MDS) method. This antigenic map implicitly implies the distance between antigen and antiserum with unknown HI titer. Cai et al. first recovered the normalized HI table by a low-rank matrix completion method (Cai et al., 2010), and then calculated the antigenic distance using the fully recovery normalized HI table and mapped it into a 2D or 3D (Barnett et al., 2012) antigenic map. Imputation-based methods can better detect the antigenic evolutionary trend of H3N2 influenza virus, but it is still insufficient. For example, the accuracy of its predicted antigenic distance is yet to be improved (Huang et al., 2017).

The antigenic evolution of influenza viruses are ultimately caused by genetic changes of the viruses especially on HA and NA genes, thus principally the sequence information of antigens and antisera will help predict missing values in HI. In this study, we propose a novel algorithm called matrix completion with antigen and antiserum similarity (MCAAS), which integrates antigen sequence information and antiserum information in a low-rank matrix completion model to predict influenza antigenicity. To our best knowledge, this the first model to leverage both the low-rank space of viruses spaces and the importance of genetic mutations in predicting influenza antigenicity. To explore the influence of different amino acids properties on the prediction of the antigenicity of the H3N2 influenza virus, we systematically compared the 65 amino acid substitution matrices in the AAindex database (Shuichi Kawashima et al., 2008), reflecting a comprehensive list of amino acid properties, including structural, physicochemical, and biochemical information. In addition, in order to make full use of the information, we have proposed a mixed-rank strategy to improve the sliding window method. The algorithm proposed in this paper was applied to H3N2 influenza data from 1968 to 2003. We then constructed an antigenic map based on the fully recovered HI table and evaluated existing vaccine strains. Finally, we explored the relationship between the genetic and antigenic evolution of the influenza virus in H3N2 data.

## Materials and methods

### Dataset and problem formulation

H3N2 influenza data are used in this study (Smith et al., 2004), which is a partially revealed HI table consisting of 253 viruses (antigens) and 79 vaccine (antisera) from 1967 to 2003, i.e., a matrix of 253 rows and 79 columns. The HI table contains Type I data, Type II data, Type III data, which are regular HI titers, low reactors (i.e., the HI titers less than a threshold) and missing values (Cai et al., 2010). Similar to many literatures (Smith et al., 2004; Cai et al., 2010; Sun et al., 2013; Huang et al., 2017), the HI table was normalized to facilitate subsequent analyses. We also downloaded HA protein sequences of viruses and vaccine strains related to HI tables from the NCBI influenza database. Only the sequence on 329 sites belonging to the HA1 protein was kept for further analysis (Yao et al., 2017). We also downloaded 65 amino acid substitution matrices from the AAindex database (Shuichi Kawashima et al., 2008) to analyze the effect of amino acid structure, physical and biological information on predicting influenza antigenicity. In this paper, the problem is how to accurately estimate low reactors and predict missing values based on values on regular entries and fusion information, which combines multiple amino acids substitution matrices and sequence information of the viruses and vaccine strains.

### Matrix completion with antigen and antiserum similarity

In this paper, we consider the problem of predicting the antigenicity of influenza viruses against vaccines, which is to fill the missing values in the HI table (as well as corrections for Type I and Type II data). Without considering the temporal bias effect, we can convert this problem into a matrix completion problem (Cai et al., 2010). Specifically, we use H to denote an HI table with m rows and n columns, which corresponds to m antigens and n antisera. Let E to represent the corresponding Type I and Type II data locations in H. Let X be the underlying matrix to recover H. Since X is in a low-dimensional space for influenza viruses, we assume that r ≪ min(*m, n*) as the rank of X. For some Σ_*r*×*r*_ matrices, X can be expressed as $\text{X}=U_{m\times r}\Sigma_{r\times r}{\left(V_{n\times r}\right)}^{T}\;$according to singular value decomposition.

In the literature (Huang et al., 2017), it has been shown that incorporating the HA protein sequence information of viruses into the matrix completion method achieves better results. However, since the model does not use vaccine strains HA protein sequence information, the use of information is incomplete. Moreover, the calculation of protein sequence similarity in this model does not take into account the physicochemical and biochemical properties of amino acids. In addition, the effect of the antigenic determinant regions on protein properties was not discussed in detail. Therefore, in order to solve the above deficiencies, we propose two new models that incorporate the above information into the matrix completion model.

Model 1 without Type II data

$$\begin{array}{l} {\text{Min}}_{X}\frac{1}{2}\overset{m}{\underset{i=1}{\sum }}\overset{n}{\underset{j=1}{\sum }}{\left(X_{ij}^{E}-H_{ij}^{E}\right)}^{2}+\lambda_{1}G\left(X\right)+\lambda_{2}\overset{m-1}{\underset{i=1}{\sum }}\overset{m}{\underset{j=i+1}{\sum }}K_{ij}\\ ||X^{i}-X^{j}|{|}^{2}+{\;\lambda }_{3}\overset{n-1}{\underset{i=1}{\sum }}\overset{n}{\underset{j=i+1}{\sum }}T_{ij}||{\left(X^{T}\right)}^{i}-{\left(X^{T}\right)}^{j}|{|}^{2} \end{array}$$

Model 2 with Type II data

$$\begin{array}{l} {\text{Min}}_{X}\frac{1}{2}\overset{m}{\underset{i=1}{\sum }}\overset{n}{\underset{j=1}{\sum }}{\left(X_{ij}^{E}-H_{ij}^{E}\right)}^{2}I\left(X_{ij}^{E}\geq \theta_{ij}\right)+\lambda_{1}G\left(X\right)+\lambda_{2}\\ \overset{m-1}{\underset{i=1}{\sum }}\overset{m}{\underset{j=i+1}{\sum }}K_{ij}||X^{i}-\;X^{j}|{|}^{2}+\lambda_{3}\overset{n-1}{\underset{i=1}{\sum }}\overset{n}{\underset{j=i+1}{\sum }}T_{ij}||{\left(X^{T}\right)}^{i}-{\left(X^{T}\right)}^{j}|{|}^{2} \end{array}$$

The function $G\left(X\right)=\overset{m}{\underset{i=1}{\sum }}g\left(\frac{||U^{i}|{|}^{2}}{3\delta r}\right)+\overset{n}{\underset{i=1}{\sum }}g\left(\frac{||V^{i}|{|}^{2}}{3\delta r}\right)\;$ is a regularization term, where *g*(*z*) = *e*^(*z*−1)^2^^ − 1 when *z* ≥ 1 and *g*(*z*) = 0, otherwise. *U*^*i*^ and *V*^*i*^ denote the *ith* row of *U* and *V*, respectively and δ = max(*m, n*) (Keshavan et al., 2009a; Cai et al., 2010). *K*_*ij*_ is the HA protein sequence similarity between virus i and j, *T*_*ij*_ is the HA protein sequence similarity between vaccine strains for vaccine i and j. *X*^*i*^ and *X*^*j*^ represent the *ith* row and *jth* row of X, respectively. (*X*^*T*^)^*i*^ and (*X*^*T*^)^*j*^ represent the *ith* column and *jth* column of *X*, respectively. The three parameters λ_1_, λ_2_, and λ_3_ control the contribution of matrix completion, HA1 protein sequence of antigens and HA1 protein sequence of vaccine strains to recover the matrix. The third and fourth terms in the model are based on the assumption that if the viruses (vaccine strains) have similar HA protein sequences (especially in antigenic determinant regions), they should have similar HI titers against the same group of vaccines (viruses). Based on previous literatures, the antigenic regions B and C seems to be more important than A, D, and E (Yao et al., 2017). Thus, we define $K_{ij}=\xi_{1}K_{ij}^{ADE}+\xi_{2}K_{ij}^{BC}+K_{ij}^{other}$($T_{ij}=\xi_{1}T_{ij}^{ADE}+\xi_{2}T_{ij}^{BC}+T_{ij}^{other}$) as the similarity calculation formula, in which ξ_1_ and ξ_2_ are the parameter to control the weight of antigenic determinants. $K_{ij}^{ADE}$ measures sequence similarity on antigenic determinant regions site A, site D, and site E. $K_{ij}^{BC}$ measures sequence similarity on antigenic determinant regions site B and site C. $K_{ij}^{other}$ measures sequence similarity on other site. Parameters λ_1_, λ_2_, λ_3_, ξ_1_, and ξ_2_ were tuned by 10-fold cross-validation.

### An alternating gradient descend method

To solve Model 1, we propose an alternating gradient descend AGD method similar to literature (Keshavan et al., 2009a; Cai et al., 2010). Since the corresponding singular vectors are highly concentrated on the high-weight row (column) index when |*E*| = Θ(*n*) (Keshavan et al., 2009b), in order to ensure that the number of non-zero values per row (column) is less than $\frac{2|E|}{m}$($\frac{2|E|}{n}$), we need to trim the H matrix. When a row (column) has more non-zero values than $\frac{2|E|}{m}$($\frac{2|E|}{n}$), we randomly set some non-zero values to zero.

We replace all missing values in *H* with 0 to form *H*^(0)^. After singular value decomposition (SVD) *H*^(0)^ = *UΣV*^*T*^, we set $U^{\left(0\right)}=U_{0}*\sqrt{m}$ and $V^{\left(0\right)}=V_{0}*\sqrt{n}$ as initial values, where, *U*_0_ and *V*_0_ consist of the first r columns of *U* and *V*, respectively.

Then we use the following updates until convergence or reaching a preset maximum number of iterations.

Fix *U*^(*t*)^ and *V*^(*t*)^ and calculate the matrix Σ_*r*×*r*_ to minimize the Model 1 as follows:

$\begin{array}{l} vec(\Sigma_{r\times r})=(V^{T}V⊗V^{T}H^{T}V+\lambda_{2}V^{T}V⊗U^{T}K_{L}^{T}U+\lambda_{3}\\ {U^{T}U⊗V^{T}T_{L}^{T}V)}^{-1}vec(U^{T}HV) \end{array}$

where ⊗ is Kronecker Product, *K*_*L*_ is the Laplacian matrix of K and *T*_*L*_ is the Laplacian matrix of T.

Update *U*^(*t*+1)^ and *V*^(*t*+1)^ using gradient descent: *U*^(*t*+1)^ = *U*^(*t*)^ + α∇*U*^(*t*)^ and *V*^(*t*+1)^ = *V*^(*t*)^ + α∇*V*^(*t*)^.

The gradients of U and V are:

$$\begin{array}{l} \nabla U=((U\Sigma V^{T})-H)V\Sigma^{T}+UQ_{U}+\lambda_{1}f(U,2e^{{\left(Q_{U1}-I_{1}\right)}^{2}}\\ \;(Q_{U1}-I_{1}))+4\lambda_{2}AU\Sigma (V^{T}V)\Sigma^{T}+4\lambda_{3}(U\Sigma V^{T})BV\Sigma^{T}\\ \nabla V={\left((U\Sigma V^{T})-H\right)}^{T}U\Sigma +VQ_{V}+\lambda_{1}f(V,2e^{{\left(Q_{V1}-I_{2}\right)}^{2}}\\ \;(Q_{V1}-I_{2}))+4\lambda_{2}V\Sigma^{T}U^{T}AU\Sigma +4\lambda_{3}BV\Sigma^{T}(UTU)\Sigma \end{array}$$

Where *A* = [_*a*_*ij*_]*m*×*m*_ and *B* = [_*b*_*ij*_]*n*×*n*_ are symmetric matrix with $a_{ij}=\{\begin{array}{c} \Sigma_{h\neq i}K_{ih}\;if\;i=j\\ -K_{ij}\;if\;i\neq j \end{array}$ and $b_{ij}=\{\begin{array}{c} \Sigma_{h\neq i}T_{ih}\;if\;i=j\\ -T_{ij}\;if\;i\neq j \end{array}$

$$\begin{array}{l} Q_{U}=\frac{1}{m}U^{T}\left(\left(H-\left(U\Sigma V^{T}\right)\right)\right)V\Sigma^{T},\\ Q_{V}=\frac{1}{n}V^{T}{\left(W{}^{\circ}\left(H-\left(U\Sigma V^{T}\right)\right)\right)}^{T}U\Sigma \\ I_{1}={\left(1,1,\ldots 1\right)}_{m\times 1}^{T},I_{2}={\left(1,1,\ldots 1\right)}_{n\times 1}^{T},\\ Q_{U1}=\frac{1}{3\alpha r}\left[\begin{array}{c} \overset{r}{\underset{j=1}{\sum }}U_{1j}^{2}\\ \overset{r}{\underset{j=1}{\sum }}U_{2j}^{2}\\ \vdots \\ \overset{r}{\underset{j=1}{\sum }}U_{mj}^{2} \end{array}\right];Q_{V1}=\frac{1}{3\alpha r}\left[\begin{array}{c} \overset{r}{\underset{j=1}{\sum }}V_{1j}^{2}\\ \overset{r}{\underset{j=1}{\sum }}V_{2j}^{2}\\ \vdots \\ \overset{r}{\underset{j=1}{\sum }}V_{nj}^{2} \end{array}\right] \end{array}$$

Where α = max(*m, n*) and *f*(*C*_*m*×*r*_, *D*_*m*×1_) = *Z*_*m*×*r*_ with

$$\begin{array}{l} Z_{ij}=\;\{\begin{array}{ll} \frac{1}{\alpha r}C_{ij}*D_{i} & if\;D_{i}>0\\ 0 & otherwise \end{array} \end{array}$$

The difference between Model 2 and Model 1 is that Model 2 has Type II data, where the Type II data is treated differently by multiplying B in the model. Therefore, we use the same method to solve Model 1 and Model 2. We only need to replace ${\sum \sum \left(X_{ij}^{E}-H_{ij}^{E}\right)}^{2}$ with $\sum \sum {\left(X_{ij}^{E}-H_{ij}^{E}\right)}^{2}I\left(X_{ij}^{E}\geq \theta_{ij}\right)$ and replace ((*UΣV*^*T*^)^*E*^ − *H*^*E*^) with ((*UΣV*^*T*^)^*E*^ − *H*^*E*^)·I. Here, I is an indication matrix, dot multiplication denotes the multiplication of corresponding elements between the matrices.

### A sliding window method

**Figure 1:**
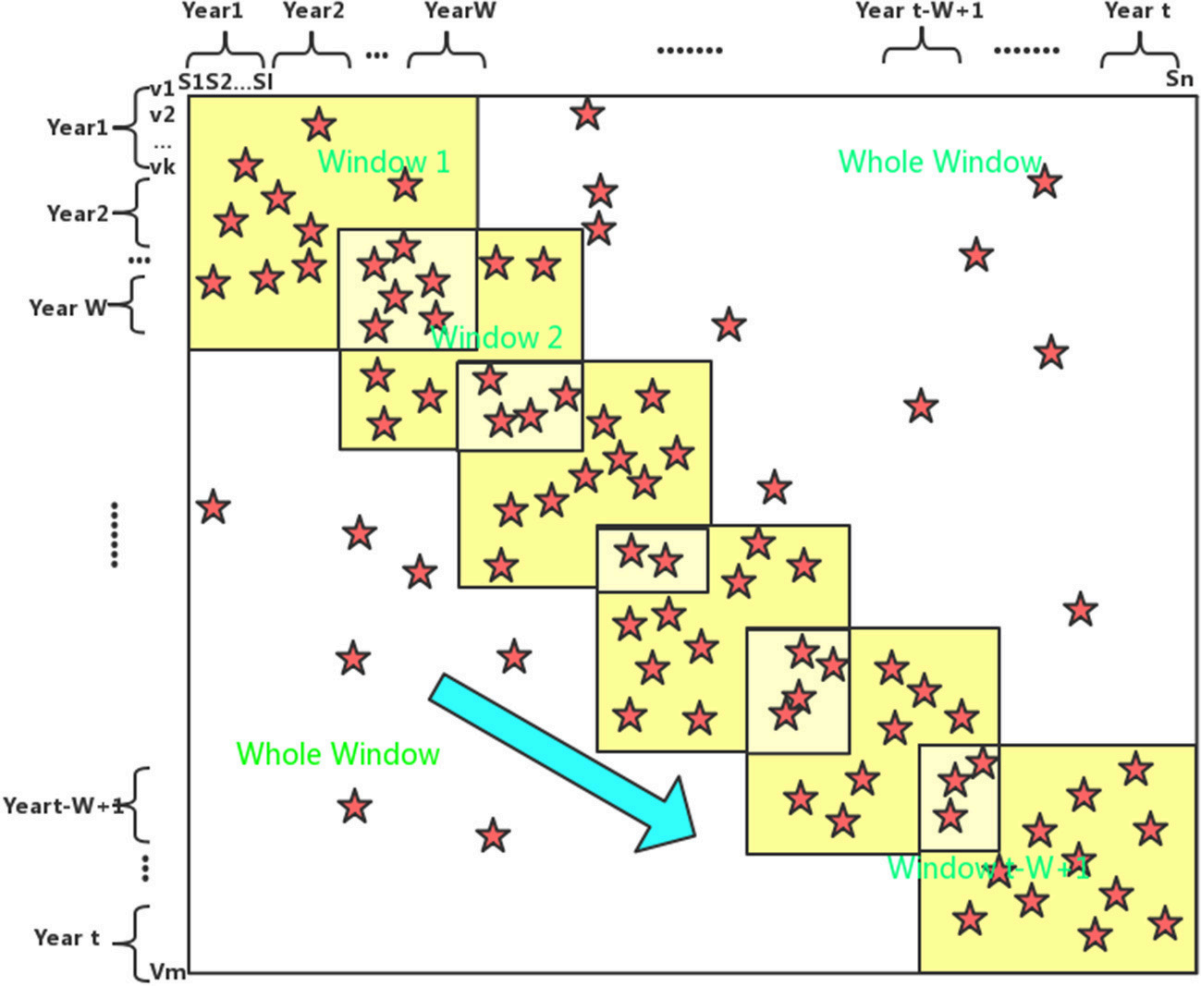
A cartoon to show the sliding window process. Row and column indicate antigen and antiserum, respectively, which are placed chronologically from the up-left to bottom-right of the window. MCAAS is used from the first window consisting of antigens and antisera starting from Year1 to the t-w+1 window consisting of antigens and antisera starting from Year t-w+1. In the shaded region, the final values are taken as the mean of the completed values in all related windows. In the end, MCAAS is performed on the whole window.

Since there is temporal bias in the HI matrix that can affect the accuracy of the matrix completion, in this paper we introduce a sliding window method to reduce this effect. The method mainly based on the principle that the temporal bias effect becomes smaller in the temporal-grouped submatrix than in the entire HI matrix. The generally flow of the method is summarized in Figure 1: let *Y*_0_ and *Y* be the starting and ending year and *W* be the window size. Then the *i* + 1*th* window year span should be from (*Y*_0_ + *i*) to (*Y*_0_ + *i* + *W*) and there is a total of (*Y*−*W* + 1) windows. Since the rank of the submatrix is less than or equal to the rank of the full-matrix, it is reasonable to consider a mixed rank rather than a single rank consistent with the full-matrix. In this paper, the rank of the submatrix is set to *rank*′ and (*rank*′−1) in the window sliding method, and *rank*′ is the setting of the full-matrix rank. Missing values are estimated on each submatrix in the case of rank *rank*′ and (*rank*′−1), and then the average of these estimates is taken as the recovered value of the corresponding position of the matrix. After the window is sliding, a partially recovered HI matrix is obtained, and on the basis of this, the algorithm proposed in this paper is performed on the whole window to fill in the values that has not been recovered.

### Performance evaluation

The performance of imputation algorithms is evaluated using the root-mean-squared error (RMSE). Given *k* values {*O*_1_, *O*_2_, …, *O*_*k*_} and {*P*_1_, *P*_2_, …, *P*_*k*_}, the RMSE is defined as:

(1) $$\begin{array}{l} \text{RMSE}=\sqrt{\frac{\sum_{i=1}^{k}{\left(O_{i}-P_{i}\right)}^{2}}{k}} \end{array}$$

Where *O*_*i*_ represents an observed value and *P*_*i*_ represents the corresponding predicted value. The smaller the RMSE value is, the closer the predicted value is to the observed value, indicating that the performance of the algorithm is better.

In this paper, we use 10-fold cross validation to calculate the RMSE value. Specifically, the H matrix is randomly divided into 10 equal parts. We will run it repeatedly for 10 times in the experiment until each part was used as the prediction set once. Each time, we use 9 parts for matrix completion; then calculate the RMSE between the completed matrix and the observed matrix entry in the remaining part. The mean RMSE between the predicted values and observed values across 10 runs are used to compare different methods. And the model parameters λ_1_, λ_2_, λ_3_, ξ_1_, ξ_2_, *r*, and *w* are tuned in this process.

### Construction of antigenic and genetic cartography

Similar to literature (Cai et al., 2010; Barnett et al., 2012), we use the Euclidean distance between viruses after completion of the matrix as the antigenic distance. Then, multidimensional scaling (MDS) is used to generate virus coordinates and construct antigen maps based on antigenic distances. The construction of the genetic map is similar to the antigenic map. We first calculate the P-distance matrix between pairs of viruses and use MDS to construct the genetic map.

## Results

### Dataset

In this paper, we used the H3N2 influenza data as our test dataset for HI values from 253 viruses against 79 antisera. There are 3,991 observed HI values in this matrix, and the sparseness is about 0.2. These viruses formed 11 antigenic clusters, namely HK68, EN72, VI75, TX77, BK79, SI87, BE89, BE92, WU95, SY97, and FU02 (Smith et al., 2004). The HA1 protein sequences of viruses and vaccine strains were then downloaded from the NCBI Influenza Virus Database.

### Matrix completion for HI table of H3N2

In this paper, we used the similarity matrix between protein sequences to assist matrix completion. There are many amino acid substitution matrices that reflect different amino acid properties, and the literatures (Lee and Chen, 2004; Liao et al., 2008) show that the substitution matrix is critical to the accuracy of the prediction. To investigate the effect of different amino acid properties on the evolution of antigens, we used the method in this article to evaluate 65 amino acid substitution matrices with parameters set to ξ_1_ = 500, ξ_2_ = 1000, *r* = 10, *w* = 32, lam1 = 1E-4, and lam2 = lam3 = 2.5E-7 (after normalizing the similarity matrix). The 10-fold cross-validation root mean square errors (RMSE) for all 65 substitution matrices were presented in Supplementary Table S1, with the top 12 RMSEs summarized in Table 1.

**Table 1:** The top 12 amino acids substitution matrices in predicting influenza antigenicity.

**Accession number**	**Description**	**RMSE**
PRLA000102	Homologous structure derived matrix (HSDM) for alignment of distantly related sequences	0.6349
HENS920101	BLOSUM45 substitution matrix	0.6351
JOND920103	The 250 PAM PET91 matrix	0.6352
QU_C930101	Cross-correlation coefficients of preference factors main chain	0.6352
PRLA000101	Structure derived matrix (SDM) for alignment of distantly related sequences	0.6352
KANM000101	Substitution matrix (OPTIMA) derived by maximizing discrimination between homologs and non-homologs	0.6352
CSEM940101	Residue replace ability matrix	0.6353
LUTR910107	Structure-based comparison table for other class	0.6354
MIYS930101	Base-substitution-protein-stability matrix	0.6354
BENS940104	Genetic code matrix	0.6355
NIEK910102	Structure-derived correlation matrix 2	0.6355
HENS920103	BLOSUM80 substitution matrix	0.6358

As can be seen from Table 1, different substitution matrices have a certain influence on the prediction result. The best substitution matrix is “Homologous structure derived matrix (HSDM) for alignment of distantly related sequences.” The RMSE obtained by using it is 0.6349. This implies the importance of HA1 protein structure in influenza antigenicity, since the best ones are based on structure-based substitution matrices, which is very reasonable because the structural information is the key to the binding affinity between the antigen and the antisera (Hirst, 1943).

We set the mixed low-rank r to vary from 6 to 14, and the sliding window size W to vary from 8 to 32 with a step size of 4. Here we used the PRLA000102 substitution matrix to measure sequence similarity and use the mixed rank window sliding method proposed in this paper. Other parameters are consistent with before. We listed the 10-fold cross validation RMSEs for different *r* and *W* in Table 2. As can be seen from the table, the lowest RMSE 0.5982 is achieved at window size 32 and rank 9 (mixed rank of 8 and 9). According to Huang et al. (2017), the best RMSE value for BMCSI is 0.6586 and those for antigenic cartography (Smith et al., 2004; Cai et al., 2010) and AntigenMap (Barnett et al., 2012) are 1.04 and 1.05, respectively. The above results indicate that the complete HA1 protein sequence information with discriminating antigenic determinant regions is a good compensation for low rank matrix completion. From Tables 1, 2, it is clear that a mixed rank sliding window method is more appropriate in completing the H3N2 influenza data.

**Table 2:** Ten-fold cross-validation RMSEs for different window sizes and ranks on H3N2 data.

**w\r**	**6**	**7**	**8**	**9**	**10**	**11**	**12**	**13**	**14**
8	1.2563	1.3737	1.5396	/	/	/	/	/	/
12	1.0394	1.0995	1.1398	1.1838	1.1749	1.1676	1.4021	/	/
16	0.9902	0.9802	0.9666	0.9011	0.9427	0.9909	1.1016	1.1051	1.2234
20	0.9434	0.9125	0.8681	0.9542	0.8833	0.8621	0.8765	0.9284	0.9421
24	0.8322	0.8705	0.8218	0.8648	0.7294	0.7396	0.7475	0.7722	0.8516
28	0.7939	0.6995	0.7879	0.7894	0.6792	0.6320	0.6881	0.7083	0.7958
32	0.7856	0.7294	0.6503	0.5982	0.6068	0.6872	0.7069	0.6779	0.6577

In order to analyze the dependence of the model on available virus information, we selected 3 viruses for single virus information analysis, and selected 12 viruses for combined virus information analysis. In the analysis of single virus information dependence, we selected the 10th, 130th, and 240th rows of virus information in the HI table, sequentially deleted about 10% of the information. The results of the analysis were shown in Table 3. It can be seen from Table 3 that with the increase of information deletion, the prediction performance of the model is generally declining. In the analysis of combined virus information dependence, we ran all the cases where all the individual virus information was deleted, the case of 2 virus combinations, the case of 4 virus combinations, the case of 6 virus combinations, and the case of 12 virus combinations. The results of the analysis were shown in Table 4. As can be seen from Table 4, as more virus information is deleted, the prediction effect becomes worse and worse.

**Table 3:** Ten-fold cross-validation RMSEs for the analysis of single virus information dependence on H3N2 data.

**row**	**10%**	**20%**	**30%**	**40%**	**50%**	**60%**	**70%**	**80%**	**90%**	**100%**
10	0.6028	0.5985	0.6052	0.5966	0.6288	0.6385	0.6427	0.6410	0.6268	0.6585
130	0.6049	0.6360	0.6182	0.6583	0.6453	0.6693	0.6746	0.6171	0.6157	0.7115
240	0.5803	0.5762	0.5862	0.5886	0.5933	0.6115	0.6176	0.6405	0.6438	0.7651

*“row” means the row where the information was deleted in HI. “10%, …, 100%” means the proportion of information deleted*.

**Table 4:** Ten-fold cross-validation RMSEs for the analysis of combined virus information dependence on H3N2 data.

**row**	**RMSEs**	**row**	**RMSEs**	**row**	**RMSEs**	**row**	**RMSEs**
10	0.6585	130	0.7115	10/30	0.7296	10/30/50/70	0.9707
30	0.6987	150	0.7874	50/240	0.8178	90/110/130/150	0.9405
50	0.6958	180	0.7282	70/110	0.8524	180/200/220/240	1.0118
70	0.7742	200	0.7666	90/220	0.7988	10/30/50/70/90/110	1.0101
90	0.7131	220	0.7280	130/180	0.7419	130/150/180/200/220/240	1.1963
110	0.7543	240	0.7651	150/200	0.8243	10/30/50/70/90/110	1.4959
						130/150/180/200/220/240	

*“row” means the row where the information is completely deleted in HI. For example, “10” and “30” mean that the 10th line and the 30th line are deleted, respectively. “10/30” means the 10th line and the 30th line are deleted at the same time*.

### Antigenic cartography for H3N2 viruses

**Figure 2:**
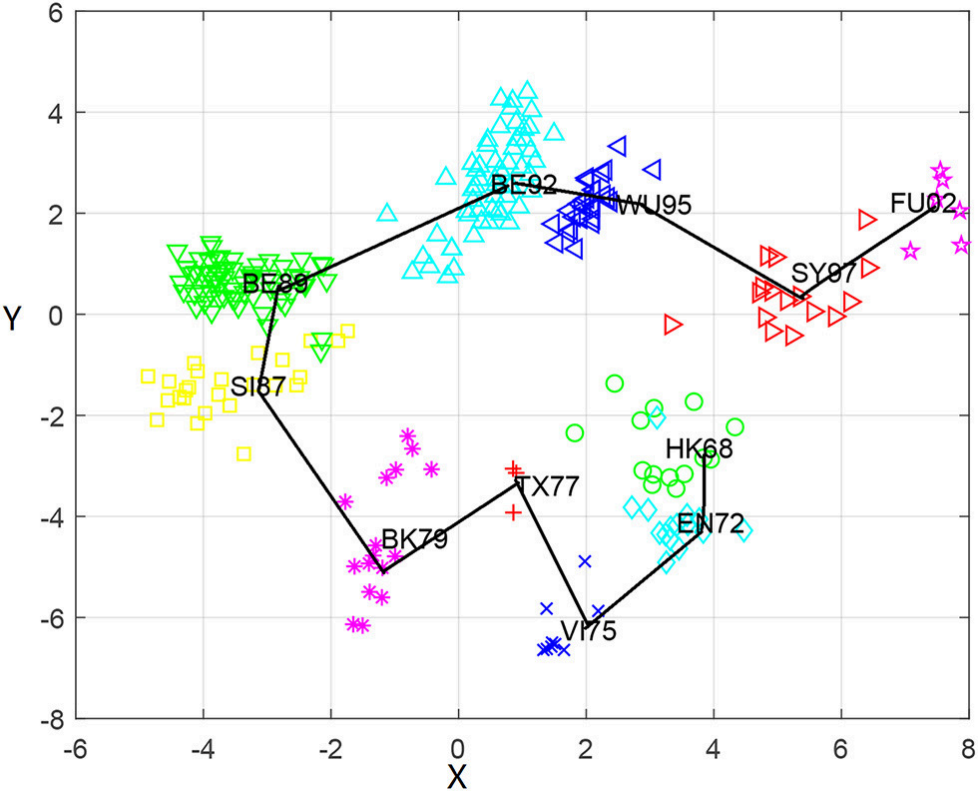
The antigenic cartography of H3N2 influenza viruses from 1968 to 2003 constructed by MCAAS. Each node denotes a virus and the distance between two nodes reflect their antigenic distance. The viruses in 11 antigenic clusters (HK68, EN72, VI75, TX77, BK79, SI87, BE89, BE92, WU95, SY97, and FU02) are marked with different shapes and colors.

Based on the antigenic distance predicted by the MCAAS method, we constructed an antigenic map of 253 viruses in H3N2 by multidimensional scaling in Figure 2. As can be seen from Figure 2, 11 antigen clusters can be distinguished very well, especially VI75, TX77, BK79, SI87, BE89, BE92, WU95, SY97, and FU02. It is reasonable since there are more HI observations in these later years, resulting in more reliable calculations. We can also find from Figure 2 that the virus has generally evolved locally along an S-shaped pathway, which are consistent with previous research (Smith et al., 2004).

We also calculated the average antigenic distances within cluster and between clusters (see Table 5), which are generally consistent with Figure 2. For example, the antigenic distance between BE92 and BE89 is much greater than the antigenic distance between SI87 and BE89 in Figure 2, whose corresponding average antigenic distances were 4.86 and 3.23, respectively. From Table 3, we can find that the average within-cluster distances of the 11 clusters are all <1.7, and the inter-cluster distances are >1.7 except for BK79-BK79 (1.64) and BE92-BE92 (1.68). In addition, the antigenic distance between viruses becomes larger as the time interval increases.

**Table 5:** The average antigenic distances among viruses within and between 11 antigenic clusters for H3N2 influenza.

	**HK68**	**EN72**	**VI75**	**TX77**	**BK79**	**SI87**	**BE89**	**BE92**	**WU95**	**SY97**	**FU02**
HK68	1.29	1.97	5.00	4.41	6.48	7.27	6.83	4.80	6.15	5.55	7.00
EN72	1.97	0.68	2.12	3.18	6.45	7.68	7.57	6.52	7.01	5.04	8.18
VI75	5.00	2.12	0.52	1.94	3.71	8.49	7.07	10.48	7.77	6.57	16.95
TX77	4.41	3.18	1.94	0.28	1.77	5.08	6.35	4.57	4.32	6.07	11.65
BK79	6.48	6.45	3.71	1.77	1.64	4.44	6.00	6.62	4.51	8.70	16.96
SI87	7.27	7.68	8.49	5.08	4.44	1.40	3.23	4.61	7.14	7.98	12.39
BE89	6.83	7.57	7.07	6.35	6.00	3.23	0.88	4.86	5.21	7.01	9.96
BE92	4.80	6.52	10.48	4.57	6.62	4.61	4.86	1.68	2.55	6.19	8.03
WU95	6.15	7.01	7.77	4.32	4.51	7.14	5.21	2.55	0.85	3.76	5.79
SY97	5.55	5.04	6.57	6.07	8.70	7.98	7.01	6.19	3.76	1.22	2.32
FU02	7.00	8.18	16.95	11.65	16.96	12.39	9.96	8.03	5.79	2.32	1.13

### Relationship between influenza genetic and antigenic evolution of H3N2

**Figure 3:**
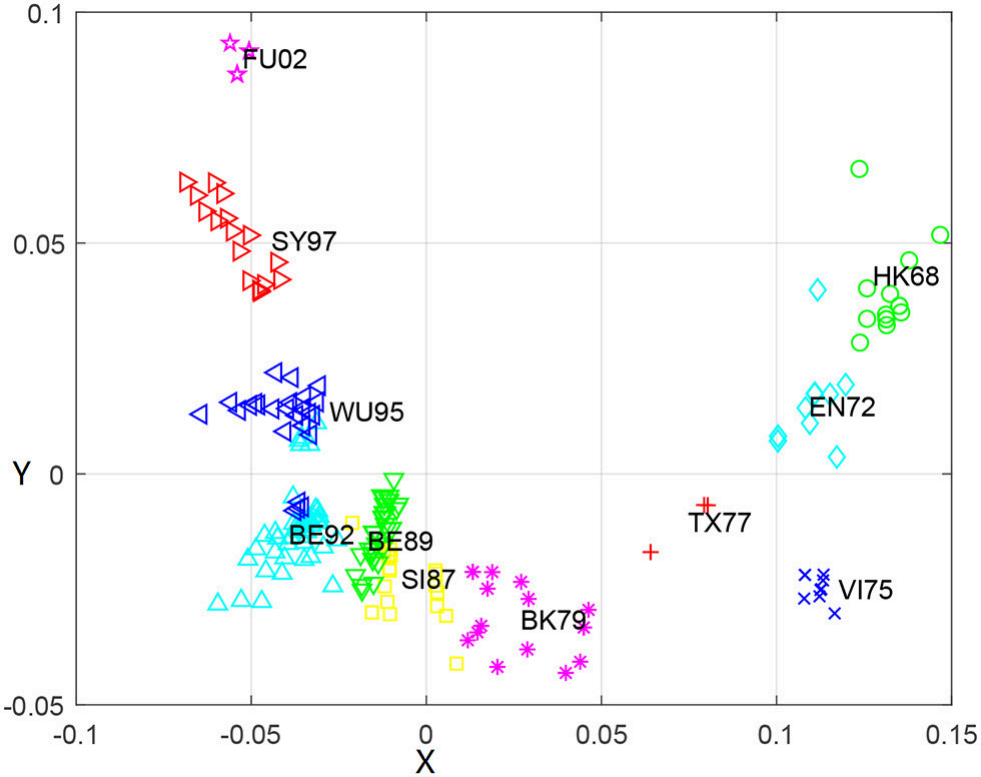
The genetic map using the uncorrected-P distance for HA1 protein sequences of H3N2 influenza virus from 1968 to 2003. Each node denotes a virus and the distance between two nodes reflect their genetic distance. The viruses in 11 antigenic clusters (HK68, EN72, VI75, TX77, BK79, SI87, BE89, BE92, WU95, SY97, and FU02) are marked with different shapes and colors.

To further explore relationship between the genetic and antigenic evolution of the H3N2 virus, we not only constructed a genetic map (Figure 3) of the 253 viruses using the uncorrected P-distance and MDS, but also calculated the average genetic distance (uncorrected P distance) of viruses within and between 11 antigen clusters (Table 6). As shown in Figure 3, it can be seen that the genetic evolution of the virus proceeds along a semicircle. By comparing Figure 2 with Figure 3, we found that the genetic and antigenic profiles are partially consistent. However, their evolutionary shapes are different, and genetic maps are more continuous, while antigenic maps are more punctual. From Table 4, we can see that the genetic distance between clusters increases with the increase of time span. The average genetic distance within-cluster varies from 0.004 to 0.025, while the average genetic distance between-clusters varies from 0.025 to 0.165.

**Table 6:** The average genetic distances among viruses within and between 11 antigenic clusters for H3N2 influenza.

	**HK68**	**EN72**	**VI75**	**TX77**	**BK79**	**SI87**	**BE89**	**BE92**	**WU95**	**SY97**	**FU02**
HK68	0.022	0.04	0.077	0.074	0.113	0.13	0.132	0.143	0.138	0.157	0.165
EN72	0.043	0.02	0.051	0.047	0.089	0.11	0.116	0.126	0.125	0.14	0.15
VI75	0.077	0.05	0.007	0.044	0.084	0.109	0.118	0.126	0.13	0.144	0.154
TX77	0.074	0.05	0.044	0.011	0.056	0.08	0.094	0.098	0.105	0.127	0.138
BK79	0.113	0.09	0.084	0.056	0.024	0.042	0.057	0.07	0.085	0.109	0.12
SI87	0.13	0.11	0.109	0.08	0.042	0.015	0.025	0.042	0.059	0.085	0.109
BE89	0.132	0.12	0.118	0.094	0.057	0.025	0.012	0.043	0.05	0.074	0.101
BE92	0.143	0.13	0.126	0.098	0.07	0.042	0.043	0.021	0.037	0.072	0.099
WU95	0.138	0.13	0.13	0.105	0.085	0.059	0.05	0.037	0.022	0.057	0.085
SY97	0.157	0.14	0.144	0.127	0.109	0.085	0.074	0.072	0.057	0.025	0.051
FU02	0.165	0.15	0.154	0.138	0.12	0.109	0.101	0.099	0.085	0.051	0.004

Although the genetic map and the antigenic map are roughly consistent, we also found that some viruses are very close in the genetic map, but are far apart in the antigenic map. For example, BE89 and BE92 are very close in the genetic map (Figure 3) with the average genetic distance only 0.043, but they are far in the antigenic map (Figure 2) with the average antigenic distance 4.86. This shows that not all genetic changes are equivalent to cause antigenic changes and different protein sites contribute differently to antigenic evolution (Smith et al., 2004; Lee et al., 2016).

## Discussions

It is known that the antigenicity of influenza virus changes very quickly. To prevent influenza outbreaks caused by changes in influenza virus antigens, the 80 WHO collaborating laboratories actively monitored the influenza viruses to determine vaccine strains for the next flu season. However, the selection of influenza vaccine strains is a labor-intensive and time-consuming process that relies on the identification of antigenic variants. In this paper, we propose a new method for integrating similarity information between viruses and between vaccines into matrix completion. The completed matrix was also used for constructing antigenic map, which helps to select vaccine strains.

With the development of sequencing technology, the acquisition of sequence information becomes easier. In the literature (Huang et al., 2017), it is shown that the integration of sequence information contributes to the prediction of viral antigenicity. This paper further explores the effect of fusion of sequence information on the prediction of virus antigenicity, mainly from four perspectives. (1) The integration of sequence information improved antigenic prediction. Not only the similarity information of the virus sequences but also the similarity information of the vaccine strains was used. (2) We discussed in more detail the influence of antigenic determinant regions on antigenic changes and further analyzed the B and C regions in the five antigenic determinant regions. (3) We analyzed 65 substitution matrices, which reflect the different physicochemical and biochemical properties of amino acids. The results show that the characteristics of the structure have a greater impact on antigen evolution. (4) We proposed a mixed rank sliding window method that can solve matrix completion problems more reasonably than single rank methods. As a result, our method reduces the prediction RMSE compared with the literature (Huang et al., 2017) and previous interpolation methods (Smith et al., 2004; Cai et al., 2010). On this basis, we also discovered a semi-circular genetic evolution and S-shaped antigen evolution, which is consistent with previous findings (Smith et al., 2004; Fouchier et al., 2010).

It is worth noting that although we used the H3N2 data in this paper, our method is applicable to all influenza subtype data such as H1N1, H5N1, and H7N9. In fact, this method could be applied to any data with a response matrix and predictive characteristics, such as the prediction of diseases and drugs, the association between miRNAs with diseases, and the recognition of protein folds (Wei and Zou, 2016). For example in drug-response prediction, the entries in the matrix represent the effect of drugs on samples, which can be formulated as a typical matrix completion problem. We believe that the similarity among drugs based on their chemical properties and the samples genetic and gene expression similarity will also help to infer drug effects.

## Availability

The program and data used in this study is publically available at: https://github.com/aibotina/MCAAS.git.

## Author contributions

PW developed the main method, designed and implemented the experiments, analyzed the result, and wrote the paper. WZ developed the method, and designed and implemented the experiments. BL conceived the project, and developed the method. LC conceived the project, and analyzed the result. LP designed and implemented the experiments, and analyzed the result. JY conceived the project, developed the main method, analyzed the result, and modified the paper.

### Conflict of interest statement

The authors declare that the research was conducted in the absence of any commercial or financial relationships that could be construed as a potential conflict of interest.
